# Mechanism of action of tranexamic acid in bleeding trauma patients: an exploratory analysis of data from the CRASH-2 trial

**DOI:** 10.1186/s13054-014-0685-8

**Published:** 2014-12-13

**Authors:** Ian Roberts, David Prieto-Merino, Daniela Manno

**Affiliations:** Clinical Trials Unit, London School of Hygiene & Tropical Medicine, Keppel Street, London, WC1E 7HT UK

## Abstract

**Introduction:**

To investigate the mechanism of action of tranexamic acid (TXA) in bleeding trauma patients, we examined the timing of its effect on mortality. We hypothesised that if TXA reduces mortality by decreasing blood loss, its effect should be greatest on the day of the injury when bleeding is most profuse. However, if TXA reduces mortality via an anti-inflammatory mechanism its effect should be greater over the subsequent days.

**Methods:**

Exploratory analysis, including per-protocol analyses, of data from the CRASH-2 trial, a randomised placebo controlled trial of the effect of TXA on mortality in 20,211 trauma patients with, or at risk of, significant bleeding. We examined hazard ratios (HR) and 95% confidence intervals for all-cause mortality, deaths due to bleeding and non-bleeding deaths, according to the day since injury. The CRASH-2 trial is registered as ISRCTN86750102 and ClinicalTrials.gov NCT00375258.

**Results:**

The effect of TXA on mortality is greatest for deaths occurring on the day of the injury (HR all-cause mortality = 0.83, 0.73 to 0.93). This survival benefit is only evident in patients in whom treatment is initiated within 3 hours of their injury (HR ≤3 hours = 0.78, 0.68 to 0.90; HR >3 hours = 1.02, 0.76 to 1.36). Initiation of TXA treatment within 3 hours of injury reduced the hazard of death due to bleeding on the day of the injury by 28% (HR = 0.72, 0.60 to 0.86). TXA treatment initiated beyond 3 hours of injury appeared to increase the hazard of death due to bleeding, although the estimates were imprecise.

**Conclusions:**

Early administration of tranexamic acid appears to reduce mortality primarily by preventing exsanguination on the day of the injury.

**Electronic supplementary material:**

The online version of this article (doi:10.1186/s13054-014-0685-8) contains supplementary material, which is available to authorized users.

## Introduction

The CRASH-2 trial has shown that administration of tranexamic acid (TXA) to bleeding trauma patients who are within 3 hours of injury, significantly reduces death due to bleeding (risk ratio (RR) = 0.72, 95% CI 0.63, 0.83) and all-cause mortality, without increasing the risk of vascular occlusive events [1,2]. Since the publication of these results, there has been debate about the mechanism of action of TXA in trauma patients. Several authors, observing that plasmin is pro-inflammatory, suggest that TXA increases survival by reducing inflammation [3-7].On the basis of results from an observational study of TXA administration in combat casualties, the investigators of the military application of TXA in trauma emergency resuscitation study (MATTERS) argued that the timing of the survival benefit with TXA suggests a mechanism other than haemostasis and hypothesised that TXA may attenuate the inflammatory response. Similarly, Neapolitano *et al*., noting the lack of a statistically significant difference in blood transfusion between TXA and placebo groups state that ‘it remains unclear whether the mortality benefit from TXA is from reversal of fibrinolysis or whether an inflammatory or immune modulation is the underlying mechanismʼ [5]. An expert committee convened by the US Department of Defense called for more research into the mechanism of action of TXA in trauma patients [6].

In response to these concerns, we conducted further analyses of the CRASH-2 trial data to examine the timing of the effect of TXA on mortality. It is generally accepted that deaths due to bleeding occur soon after the bleeding event. The Bleeding Academic Research Consortium (BARC), which was established to provide a standard definition of bleeding for use in clinical trials, noted that ‘the time interval from the bleeding event to the death should be considered with respect to likely causalityʼ, although they do not specify a particular interval [8]. The MATTERS investigators argued that beyond 48 h, bleeding is less likely to be the primary cause of death. We hypothesised that if TXA improves survival by reducing bleeding, its effect should be greatest on the day of the injury. On the other hand, if it improves survival by reducing inflammation its effect should be most apparent in subsequent days.

## Methods

### Study design and patients

The CRASH-2 trial was a randomised placebo-controlled trial of the effect of TXA on death and vascular occlusive events in adult trauma patients with, or at risk of, significant bleeding, and who were within 8 h of their injury. Patients were randomly assigned to receive TXA (loading dose 1 g over 10 minutes followed by an infusion of 1 g over 8 h) or matching placebo. The primary outcome was death in hospital within 4 weeks of injury. Cause of death was described using the following categories: bleeding, vascular occlusion (myocardial infarction, stroke, and pulmonary embolism), multi-organ failure, head injury, and other. Follow-up data were available for 99.6% of patients. The trial was conducted in 274 hospitals in 40 countries. A detailed description of the rationale, design, methods and results of the trial has been published previously [1].

### Analysis

Both per-protocol and intention-to-treat analyses were conducted. The effect of TXA on mortality was assessed using hazard ratios (HR), which give the ratio of the probability of death in the TXA and placebo group at a given time point, among patients surviving to that time point. It represents the instantaneous risk of death for an individual who has survived to that particular time point. We calculated HR for all-cause mortality, death due to bleeding and for non-bleeding deaths, on the day of the injury and for the subsequent four days. We also conducted analyses stratified by time from injury to the initiation of TXA treatment (≤3 h or >3 h). Precision was quantified using 95% CI.

To give a visual representation of the results, we prepared a heat map of the smoothed HR. We stratified patients into one-hour intervals by time from injury to treatment and estimated HR for all-cause mortality for each stratum over the first 5 days. We plotted these HR in a two-dimensional coloured heat map of nine by five cells. To reduce the impact of random variability, we smoothed the HR using a simple moving average method. The smoothed HR was a double-weighted average of itself and the neighbouring HR. The two weights were: 1) the inverse of the variance of the HR and 2) the inverse of the distance between the smoothed HR and the neighbouring HR. Distance was defined as 1 for the HR being smoothed, with +1 being added for each day and hour of separation up to a maximum of 3 units (see Additional file 1 for an example of distances).

The trial was considered and approved by the relevant ethics committees in all participating hospitals (see Additional file 2). Consent procedures at participating hospitals were established by local regulation and the appropriate ethics committees. Informed consent was obtained from patients if physical and mental capacity allowed. If patients could not give consent, proxy consent was obtained from a relative or representative. If a proxy was unavailable, then if permitted by local regulation, consent was deferred or waived. When consent was deferred or given by a proxy, the patient was informed about the trial as soon as possible and consent obtained for the use of the data collected.

## Results

**Figure 1:**
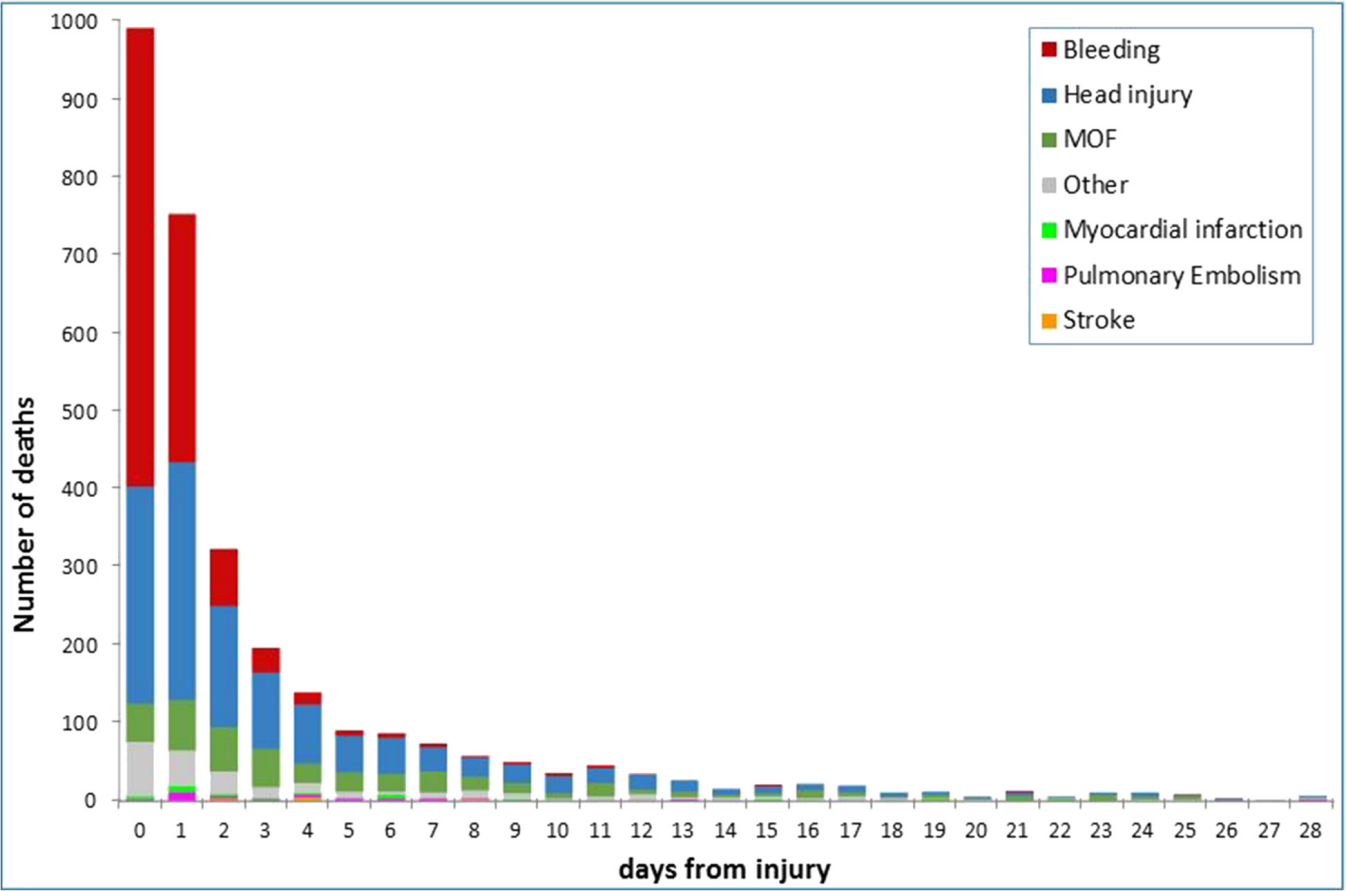
**Cause of death by day since injury.**

Of the 20,211 patients who were randomly allocated to receive TXA or placebo, primary outcome data were available for 20,127 patients (99.6%) (10,060 allocated to TXA and 10,067 to placebo). There were 3,076 deaths (15.3%). Figure 1 shows cause of death by day since injury. On the day of the injury, there were 990 (32.2%) deaths, 587 (59%) of which were due to bleeding. A total 19,944 patients (99.1%) were known to have received a loading dose of TXA or placebo and 3,021 of these patients died. The following results are based on patients who received the loading dose of TXA or placebo (per protocol analysis). The intention-to-treat analyses were essentially the same.

**Figure 2:**
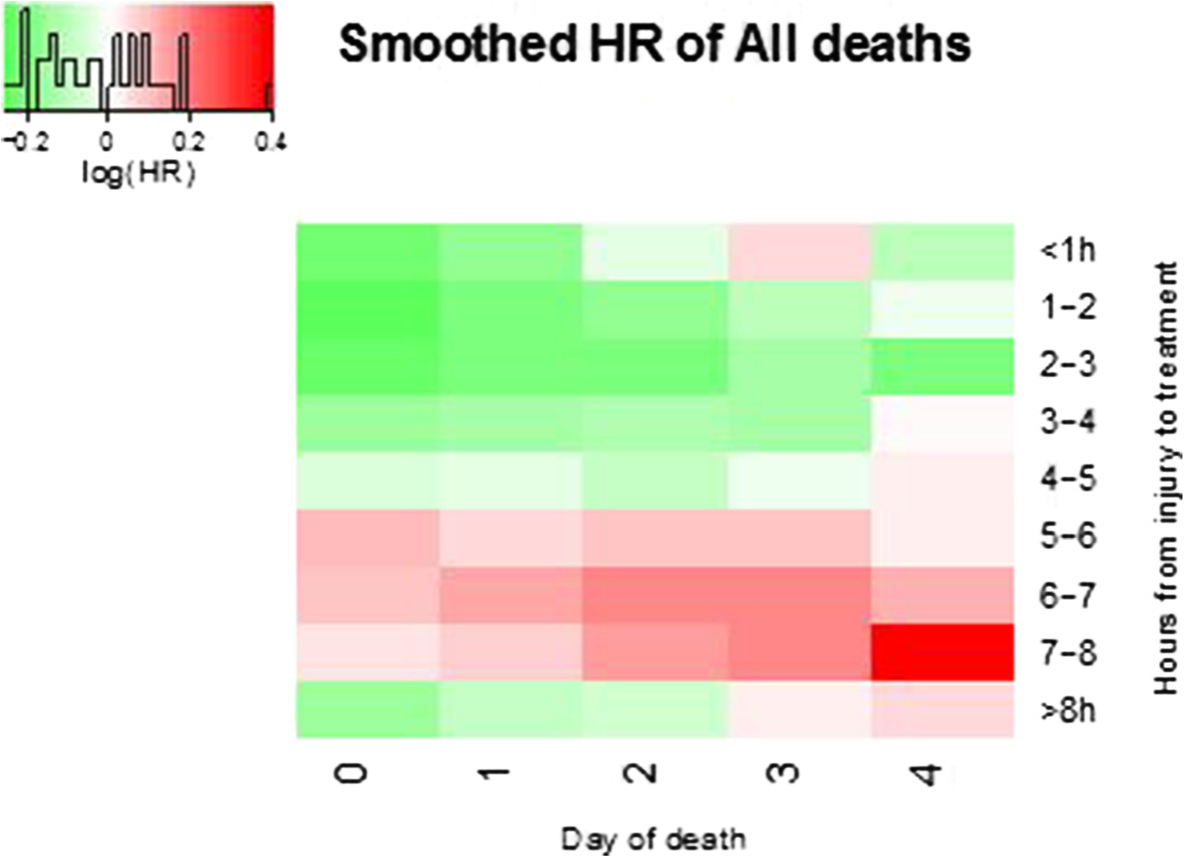
**Heat map of smoothed hazard ratios (HR) stratified by time to treatment and day of death.**

There was a significant reduction in the hazard of death from all causes in TXA treated patients on the day of the injury (HR = 0.83, 95% CI 0.73, 0.93). Thereafter, there was no significant reduction in the hazard of death from all causes (Table 1). Figure 2 shows a heat map of the smoothed HR, stratified by time from injury to treatment and day of death.

**Table 1:** **Hazard ratios (95% CI) of the effect of tranexamic acid on all-cause mortality, bleeding and non-bleeding deaths by day since injury**

**Days since injury**	**All-cause**	**Bleeding**	**Non-bleeding**
0	0.83 (0.73, 0.93)	0.80 (0.68, 0.94)	0.87 (0.71, 1.06)
1	0.91 (0.79, 1.04)	0.89 (0.72, 1.11)	0.92 (0.76, 1.11)
2	0.96 (0.77, 1.19)	1.17 (0.74, 1.86)	0.91 (0.71, 1.16)
3	1.01 (0.76, 1.34)	0.66 (0.32, 1.37)	1.09 (0.80, 1.48)
4	0.96 (0.70, 1.36)	0.77 (0.29, 2.06)	1.01 (0.71, 1.43)

Among those treated within 3 h of injury, there was a 22% reduction in the hazard of death from all causes on the day of the injury (HR = 0.78, 95% CI 0.68, 0.90). There was no reduction in the hazard of death from all causes on the day of the injury among those treated after 3 hours (HR = 1.02, 95% CI 0.76, 1.36).

There was a significant reduction in the hazard of death due to bleeding in TXA treated patients on the day of the injury (HR = 0.80, 95% CI 0.68, 0.94). Thereafter, there was no significant reduction in the hazard of death due to bleeding (Table 1). There were no significant reductions in the hazard of death from causes other than bleeding.

Table 2 shows HR for deaths due to bleeding and for non-bleeding deaths, stratified by the time interval from the injury to randomisation. For patients treated within 3 h of injury, the hazard of death due to bleeding was significantly reduced with TXA on the day of the injury (day 0), the following day (day 1) and on day 3. There were no significant reductions in the hazard of non-bleeding deaths.

**Table 2:** **Hazard ratios (95% CI) of the effect of tranexamic acid on all-cause mortality, bleeding and non-bleeding deaths by day since injury and time from injury to treatment**

**Days since injury**	**Time to treatment ≤3 h**			**Time to treatment >3 h**		
	**All-cause**	**Bleeding**	**Non- bleeding**	**All-cause**	**Bleeding**	**Non- bleeding**
0	0.78 (0.68, 0.90)	0.72 (0.60, 0.86)	0.89 (0.71, 1.11)	1.02 (0.76, 1.36)	1.28 (0.85, 1.93)	0.79 (0.51, 1.22)
1	0.86 (0.72, 1.02)	0.72 (0.55, 0.94)	0.98 (0.78, 1.23)	1.02 (0.80, 1.31)	1.47 (0.97, 2.21)	0.81 (0.59, 1.13)
2	0.86 (0.65, 1.13)	1.01 (0.58, 1.77)	0.82 (0.59, 1.12)	1.16 (0.81, 1.66)	1.61 (0.70, 3.70)	1.08 (0.72, 1.60)
3	0.95 (0.66, 1.37)	0.26 (0.09, 0.78)	1.20 (0.80, 1.81)	1.11 (0.73, 1.71)	2.76 (0.73, 10.39)	0.98 (0.62, 1.55)
4	0.94 (0.61, 1.45)	0.64 (0.18, 2.28)	0.99 (0.63, 1.57)	1.04 (0.62, 1.75)	1.04 (0.21, 5.13)	1.04 (0.60, 1.80)

## Discussion

The effect of TXA on mortality is greatest on the day of the injury, when TXA reduces the risk of death from all causes by about 20%. This survival benefit is only evident in patients in whom treatment is initiated within 3 h of injury. Thereafter, early TXA treatment has a smaller effect on all-cause mortality, although a reduction in the hazard of death due to bleeding is evident for several days. Our results suggest that there is a short time interval during which TXA administration can prevent exsanguination and that urgent treatment is therefore essential.

Whilst the benefit of early TXA treatment is greatest on the day of injury, the adverse effect of late administration manifests as an increased risk of death due to bleeding over subsequent days [2]. We have previously postulated that late initiation of TXA treatment may increase the risk of thrombotic disseminated intravascular coagulation (DIC) [9,10]. By inhibiting fibrinolysis, TXA might increase the risk of DIC. Although the underlying pathology in DIC is thrombosis, due to the consumption of coagulation factors DIC often manifests as bleeding. Among patients in whom treatment is initiated beyond 3 h of the injury, deaths apparently due to bleeding may have been due to thrombotic DIC.

The strength of this analysis is that it is based on data from a randomised controlled trial with over 20,000 patients in which there were 3,076 deaths. As a result, this study has considerable statistical power to examine the temporal effects of TXA administration. Nevertheless, because a large proportion of the deaths occurred in the first 48 h after injury, the subsequent HR are imprecise with wide confidence intervals. A further weakness is that these analyses were not pre-specified in the trial protocol with the attendant risk that random patterns in the data may have been over interpreted. These results should therefore be interpreted cautiously and in the context of other relevant research.

The hypothesis that TXA has beneficial mechanisms other than haemostasis was suggested by the MATTERS investigators on the basis that there was ‘no difference in mortality between the TXA and no TXA groups until the 48-hour pointʼ. However, the MATTERS data show a 23% relative reduction in all-cause mortality at 24 h (9.6% versus 12.4%; *P* = 0.2) [4]. Although this reduction was not statistically significant, it is not possible from these data to exclude a possible effect on mortality; rather, only that such a statistically significant effect was not observed. With much larger patient numbers, we found a similar reduction in early mortality with TXA that was highly statistically significant.

The lack of any apparent effect of TXA administration on the receipt of blood transfusion has also contributed to the debate about its mechanism of action. However, there are several possible reasons why TXA did not reduce the receipt of blood transfusion in the CRASH-2 trial. First, much of the blood loss in trauma patients occurs before hospital admission and therefore before TXA administration. Some of the transfusions that patients received in hospital would have been in response to this early bleeding and could not have been influenced by TXA. Second, because TXA reduced mortality, more TXA-treated patients had the opportunity to be transfused. Third, any effect of TXA on blood transfusion may have been diluted by transfusions given later during the hospital stay, for example transfusions given during orthopaedic surgery. Finally, accurate estimation of blood loss is difficult in trauma patients and so the amount of blood transfused might not indicate the amount of blood lost.

## Conclusions

Because coagulation, fibrinolysis and inflammation are closely interwoven pathophysiological responses, attempts to draw a clear distinction between the anti-fibrinolytic and anti-inflammatory effects of TXA may be inappropriate [11]. It would also be inappropriate to conclude that an effect on deaths prior to a specified number of hours or days necessarily implies that TXA works by reducing bleeding as opposed to reducing inflammation. Nevertheless, the finding that the effect of TXA is greatest on the day of the injury and for deaths due to bleeding supports the hypothesis that TXA improves survival by reducing bleeding. The large benefit from early administration and the absence of benefit from late administration also supports this proposition. This view is consistent with the pharmacology of TXA and with results of clinical trials of TXA in elective surgery in which blood loss can be accurately quantified [12,13]. Although the data presented here do not exclude the possibility TXA might have clinically important anti-inflammatory effects, at present there is no evidence from randomised controlled trials that TXA reduces inflammation in trauma patients, although trials in progress will provide further information [7].

## Key messages

- To investigate the mechanism of action of TXA in bleeding trauma patients we examined the timing of its effect on mortality
- We hypothesised that if TXA reduces mortality by decreasing bleeding, its effect should be greatest on the day of the injury, but if it reduces mortality via an anti-inflammatory mechanism, its effect should be greater over subsequent days
- We found that the effect of TXA on mortality is greatest on the day of the injury, when early TXA treatment reduces the risk of death from all causes by about 20% and death due to bleeding by about 30%. Our results support the hypothesis that TXA improves survival by reducing bleeding
- There is a short time-window during which TXA administration can prevent exsanguination; urgent treatment may therefore be essential

## Additional files

Additional file 1
**Distances for estimating smoothed hazard ratios.**
Additional file 2
**Local Ethics Committees.**

## Abbreviations

BARC: Bleeding Academic Research Consortium
DI: disseminated intravascular coagulation
HR: hazard ratio
MATTERS: military application of TXA in trauma emergency resuscitation study
RR: risk ratio
TXA: tranexamic acid
