# Supplementary material for: Mechanism of action of tranexamic acid in bleeding trauma patients: an exploratory analysis of data from the CRASH-2 trial
Source: Crit Care. 2014 Dec 13;18(6):685. doi: 10.1186/s13054-014-0685-8 (PMC4277654; doi:10.1186/s13054-014-0685-8)
Supplement: Additional file 1 — Distances for estimating smoothed hazard ratios. [file 13054_2014_685_MOESM1_ESM.doc]

Additional file 1

|  |  | 3 |  |  | Hours since injury |
| --- | --- | --- | --- | --- | --- |
|  | 3 | 2 | 3 |  |
| 3 | 2 | 1 | 2 | 2 |
|  | 3 | 2 | 3 |  |
|  |  | 3 |  |  |
| Days since treatment | | | | |
