# Supplementary material for: Mechanism of action of tranexamic acid in bleeding trauma patients: an exploratory analysis of data from the CRASH-2 trial
Source: Crit Care. 2014 Dec 13;18(6):685. doi: 10.1186/s13054-014-0685-8 (PMC4277654; doi:10.1186/s13054-014-0685-8)
Supplement: Additional file 2 — Local Ethics Committees. [file 13054_2014_685_MOESM2_ESM.docx]

| Local Ethics Committees |
| --- |
| AIIMS Ethics Committee |
| Airedale Local Research Ethics Committee |
| Anant Institute of Medical Sciences Ethics Committee |
| ANSH Ethics Committee |
| Apex Hospital, Bhopal Ethics Committee |
| Apollo Chennai Institutional Ethics Committee |
| Apollo Gleneagles Hospital Institutional Ethics Committee |
| Apollo Health City Ethics Committee |
| Aramada del Ecuador Hospital Naval REC |
| Argyll and Clyde Local Research Ethics Committee |
| Ayrshire and Arran Local Research Ethics Committee |
| Ayush Hospitals & Trauma Care PVT. Ltd Ethics Committee |
| Baby Memorial Hospital Ethics Committee |
| Barking and Havering Local Research Ethics Committee |
| Barnet, Enfield and Haringey Local Research Ethics Committee |
| Barnsley Local Research Ethics Committee |
| Bath Local Research Ethics Committee |
| Bedfordshire Local Research Ethics Committee |
| Berkshire Local Research Ethics Committee |
| Bexley and Greenwich Local Research Ethics Committee |
| Blackpool, Wyre and Fylde Local Research Ethics Committee |
| Bolton Local Research Ethics Committee |
| Borders Local Research Ethics Committee |
| Bradford Local Research Ethics Committee |
| Brent Medical Ethics Committee |
| Bridgend, Neath, Port Talbot and Swansea Local Research Ethics Committ |
| Brighton and Mid Sussex Research Ethics Committee B |
| Brighton East Research Ethics Committee |
| Bromley Local Research Ethics Committee |
| Brompton, Harefield and NHLI Local Research Ethics Committee |
| Burdwan Medical College Ethics Committee |
| Burirum Hospital Ethical Committee |
| Calderdale and Huddersfield Local Research Ethics Committee |
| Cambridge Research Ethics Committee |
| Camden and Islington Community Local Research Ethics Committee |
| Central and South Bristol Research Ethics Committee |
| Central India Medical Research Ethics Committee, Nagpur |
| Central Manchester Research Ethics Committee |
| Centre Hospitalier Universitaire Oran |
| Charles University in Prague |
| Cheshire Local Research Ethics Committee |
| CHL - Apollo Hospitals Ethics Committee |
| Clinical Trial Service Unit (CTSU) |
| Comissao de Etica para Analise de Projetos de Pesquisa |
| Command Hospital Ethics Committee |
| Cornwall Local Research Ethics Committee |
| County Durham and Darlington Local Research Ethics Committee |
| Coventry Local Research Ethics Committee |
| Cumbria and Lancashire Research Ethics Committee |
| Deepak Hospital Ethical Committee |
| Departmento de Docencia e Investigacion |
| Derbyshire Local Research Ethics Committee |
| Devadoss Multi Speciality Hospital Ethics Committee |
| Doncaster Local Research Ethics Committee |
| Dorset Local Research Ethics Committee |
| Dow University Of Health Sciences |
| Dudley Local Research Ethics Committee |
| Dumfries and Galloway Local Research Ethics Committee |
| Dyfed Powys Local Research Ethics Committee |
| Ealing and West London Mental Health Trust LREC |
| East and North Hertfordshire Hospitals Local Research Ethics Committee |
| East Birmingham Local Research Ethics Committee |
| East Kent Local Research Ethics Committee |
| East Lancashire Local Research Ethics Committee |
| East London and the City Research Ethics Committee |
| East Somerset Local Research Ethics Committee |
| East Surrey, Crawley and Horsham Local Research Ethics Committee |
| East Sussex Local Research Ethics Committee |
| Eastern Multi-centre Research Ethics Committee |
| EC - Aditya Diagnostics & Hospitals Institutional Ethics Committee |
| EC - Al Shifa Hospital Institutional Ethics Committee |
| EC - BGS Hospital Institutional |
| EC - Bhattacharyya Institutional Ethics Committee |
| EC - Bhumibol Adulyadej Hospital |
| EC - Clinica de Especialidades Medicas San Gregorio |
| EC - Clinica Stanta Ana Ethics Committee |
| EC - Comite de Etica Hospitalaria |
| EC - Comite de Investigaciones y Etica San Ignacio |
| EC - Empresa Social del Estado Hospital San Rafael Tunja |
| EC - Empresa Social del Estado Hospital Univ Erasmo Meoz |
| EC - ESE Hospital Universitario del Caribe |
| EC - Fundacion Clinica Valle Del Lili |
| EC - Gola Ghat Neuro Centre Ethics Committee |
| EC - Goyal Hospital Ethics Committee |
| EC - HIGA "Dr Oscar Alende" Ethics Committee |
| EC - HIGA San Martin de La Plata |
| EC - Hospital "Dr Rafael Rodriguez Zambrano" Manta |
| EC - Hospital de Ninos "Dr Roberto Gilbert Elizalde" EC |
| EC - Hospital Escuela Grail Jose de San Martin |
| EC - Hospital General "La Perla" |
| EC - Hospital General de Ecatepec "Dr Jose Ma Rodriguez |
| EC - Hospital General de Medellin |
| EC - Hospital General de Uruapan Ethics Committee |
| EC - Hospital General Ecatepec Las Americas |
| EC - Hospital Gustavo Rovirosa Perez Ethics Committee |
| EC - Hospital Jose Cayetano Heredia Ethics Committee |
| EC - Hospital Luis Vernaza Ethics Committee |
| EC - Hospital Municipal de Agudos "Dr Leonidas Lucero" |
| EC - Hospital Nacional Cayetano Heredia |
| EC - Hospital Nacional Dos de Mayo Ethics Committee |
| EC - Hospital Nacional Hipolito Unanue Ethics Committee |
| EC - Hospital Nacional Rosales Comite de Etica |
| EC - Hospital Pablo Tobon Uribe |
| EC - Hospital Padilla |
| EC - Hospital Privado Centro Medico de Cordoba |
| EC - Hospital Regional Docente de Trujillo Ethics Committee |
| EC - Hospital Regional Rio Grande |
| EC - Hospital San Andres ESE |
| EC - Hospital Universitario del Valle |
| EC - Hospital Universitario San Jose De Popayan |
| EC - Hospital Universitario San Vicente de Paul |
| EC - Hospital Univesitario Neiva Huila |
| EC - Institute of Critical Care Medicine Ethics Committee |
| EC - Lampang Hospital Institutional Review Board |
| EC - Neurosurgery Dept Tbilisi State Medical University |
| EC - Oberai Institutional Ethics Committee |
| EC - Pattani Hospital Institutional Review Board |
| EC - Ramaiah Medical College & Teaching Hospital ERB |
| EC - Rumah Sakit Umum Daerah Dr Soebandi Jember Ethics Committee |
| EC - Sancheti Institute of Orthopaedics and Rehabilitation |
| EC - Sheth VS Hospital Institutional |
| EC - Surgery Dept, Tbilisi State Medical Univ Central Clinic |
| EC - Tbilisi Central Clinic Emergency Dept |
| EC - Tbilisi State University I Javakhishvili |
| EC King George's Medical University |
| EC Krishna Surgical Hospital & Trauma Care Centre |
| EC Meenakshi Mission Hospital and Research Centre |
| EC St James Hospital |
| Empresa Social del Estado Hospital Universitario San Jorge Pereira |
| Ethical Committee (GMC Nagpur) |
| Ethical Committee of Muhammadiyah Hospital of Lamongan |
| Ethical Committee Sri Sakthi Hospital |
| Ethics Committee - Medical Trust Hospital |
| Ethics Committee BJ Medical College and Civil Hospital |
| Ethics Committee Ganga Hospital |
| Ethics Committee ISSSTE Torreon Hospital Gallindo Chavez |
| Ethics Committee King George's Medical University |
| Ethics Committee Krishna Surgical Hospital & Trauma Care Centre |
| Ethics Committee of Chettinad Academy of Research and Education |
| Ethics Committee of Christian Medical College |
| Ethics Committee of Hospital Nacional Arzobispo Loayza |
| Ethics Committee of Krishna Institute of Medical Sciences |
| Ethics Committee of Manipal Hospital & Manipal Heart Foundation |
| Ethics Committee of Nizam's Institute of Medical Sciences |
| Ethics Committee of NSCB Medical College |
| Ethics committee of Shanghai Renji Hospital |
| Ethics Committee, Gauhati Medical College Hospital |
| Ethics Committee, Government District Hospital Udhampur |
| Ethics Committee, International Hospital |
| Ethics Committee, KLE University |
| Ethikkommission des Landes Oberösterreich |
| Etická komise FN Motol |
| European Society for Emergency Medicine |
| Eyvaz Zadeh Hospital |
| Facutad de Medicina y Hospital Universitario |
| Frenchay Research Ethics Committee |
| Fundacion Hospital San Jose de Buga Ethics Committee |
| Gateshead and South Tyneside Local Research Ethics Committee |
| GGH Ethics Committee, Rajkot |
| Gloucestershire Local Research Ethics Committee |
| Grampian Local Research Ethics Committee |
| Great Yarmouth and Waveney Local Research Ethics Committee |
| GSVM Medical College IEC |
| Guys Research Ethics Committee |
| Gwent Local Research Ethics Committee |
| Hammersmith, Queen Charlottes and Chelsea Research Ethics Committee |
| Harrogate Health Care NHS Trust Local Research Ethics Committee |
| Harrow Research Ethics Committee |
| Hartlepool and North Tees Local Research Ethics Committee |
| Hereford and Worcester Local Research Ethics Committee |
| Hertfordshire Local Research Ethics Committee |
| Highland Local Research Ethics Committee |
| Himalayan Institute Hospital Trust IEC |
| Hopsital Central "Dr Miguel Perez Carreno IUSS Ethics Committee |
| Hospital 4 de Junio Ethics Committee |
| Hospital Alcivar Ethics Committee |
| Hospital de Pronto Socorro de Canoas |
| Hospital General Chimalhuacan Ethics Committee |
| Hospital General del Sur "Dr Pedro Iturbe" Ethics Committee |
| Hospital General Regional 25 Ethics Committee |
| Hospital IV Essalud Huancayo Ethics Committee |
| Hospital Jose Carrasco Ateaga |
| Hospital La Caleta Ethics Committee |
| Hospital Universitario Guayaquil Comite de etica |
| Hounslow and Hillingdon Local Research Ethics Committee |
| HPSS Phase I Research Ethics Committee |
| HPSS Research Ethics Committee 1 (HPSS1) |
| HSE North Eastern Area Research Ethics Committee |
| Hull and East Riding Local Research Ethics Committee |
| Human Ethical Committee Medical College Thiruvananthapuram |
| Human Research Ethics Committee |
| Huntingdon Research Ethics Committee |
| Independent Ethics Committee (Chikitsa) |
| Independent Ethics Committee MAANAV Health Foundation |
| Independent Ethics Committee Nagpur |
| Indian Medical Association Chettinad Branch Ethics Committee |
| Institute Ethics Committee Care Hospital |
| Institute Ethics Committee Postgraduate Institute of Medical Education and Research, Chandigarh |
| Institution Ethics Committee Sushrut Hospital |
| Institutional Ethics Committee Deccan College of Medical Sciences |
| Institutional Ethics Committee Dr Jeyasekharan Medical Trust |
| Institutional Ethics Committee for Human Research |
| Institutional Ethics Committee GMC Chandigarh |
| Institutional Ethics Committee Government Medical College (Jammu) |
| Institutional Ethics Committee Government Rajaji Hospital |
| Institutional Ethics Committee Govt. Medical College Calicut |
| Institutional Ethics Committee Goyal Hospital and Research Centre |
| Institutional Ethics Committee GSVM Medical College |
| Institutional Ethics Committee Himalayan Institute Hospital Trust |
| Institutional Ethics Committee Kasturba Medical College |
| Institutional Ethics Committee Krishna Kumar Orthopaedic Hospital |
| Institutional Ethics Committee Krishnamai Medical & Research Foundation |
| Institutional Ethics Committee LTM Medical College and LTMG Hospital |
| Institutional Ethics Committee Mahakoshal Hospital |
| Institutional Ethics Committee Malankara Orthodox Syrian Church Medical College |
| Institutional Ethics Committee NKP Salve and Lata Mangeshkar Hospital |
| Institutional Ethics Committee of Kamineni Hospitals |
| Institutional Ethics Committee of Mansarovar Hospital Group |
| Institutional Ethics Committee of MKCG Medical College |
| Institutional Ethics Committee of Sanjivani Diagnostics and Hospitals |
| Institutional Ethics Committee of Satna Hospital (P) Ltd. |
| Institutional Ethics Committee of Shanti Mukand Hospital |
| Institutional Ethics Committee Rajeev Gandhi Memorial Hospital |
| Institutional Ethics Committee Sheth VS General Hospital |
| Institutional Ethics Committee Vydehi Institute of Medical Sciences & Research Centre |
| Institutional Ethics Committee, Dr Jeyasekharan Medical Trust |
| Institutional Ethics Committee, Fortis Escorts Hospital |
| Institutional Ethics Committee, GM Hospitals |
| Instituto Autonomo Hospital U de los Andes Ethics Committee |
| Instituto Nacional de Vigilancia de Medicamentos y Alimentos |
| Isle of Wight, Portsmouth and South East Hampshire Local Research Ethi |
| Jain Medical Ethics Committee |
| Joint Commission of Ethics- City Hospital #1 |
| Jordan Food and Drug Administration |
| Jupiter Hospital |
| Kasturba Medical College IEC |
| Khon Kaen Hospital Ethical Reveiw Committee for Research |
| King's College Hospital Local Research Ethics Committee |
| Kintampo Health Research Centre - Ethics Committee |
| Krishna Kumar Orthopaedic Hospital |
| Krishnamai Medical & Research Foundation |
| Lanarkshire Local Research Ethics Committee |
| Leeds East Research Ethics Committee |
| Leeds West Research Ethics Committee |
| Leicestershire Research Ethics Committee |
| Lewisham Local Research Ethics Committee |
| Life Sciences Research Office |
| Lifeline Hospital Ethics Committee |
| Lincolnshire Local Research Ethics Committee |
| Liverpool Adult Research Ethics Committee |
| London Multi-centre Research Ethics Committee |
| London-Surrey Borders Local Research Ethics Committee |
| Lothian Local Research Ethics Committee |
| LTM Medical College and LTMG Hospital |
| Lyndon Copiers Limited |
| Mahatma Gandhi Institute of Medical Sciences Ethics Committee |
| Malaysia Medical Research Ethics Committee, Ministry of Health |
| Mallikatta Ethical Committee |
| Manavata Clinical Research Institute Professional Ethics Committee |
| Maudsley Research Ethics Committee |
| Medical Ethics Committee, University of Malaya Medical Centre |
| Meenakshi Mission Hospital and Research Centre Ethics Committee |
| Metropolitan Multi-centre Research Ethics Committee |
| Mid and South Buckinghamshire Local Research Ethics Committee |
| Milad Hospital |
| Milton Keynes Local Research Ethics Committee |
| Moorfields Eye Hospital Local Research Ethics Committee |
| Morecambe Bay Research Ethics Committee |
| Muhhamadiyah Hospital of Lamongan |
| Multi-centre Research Ethics Committee for Scotland |
| Multi-centre Research Ethics Committee for Wales |
| National Health Ethics and Research Committee |
| National Hospital for Neurology and Neurosurgery and Institute of Neurology |
| Nazareth Hospital Ethics Committee |
| Nepal Medical College Research Committee |
| Newcastle and North Tyneside Research Ethics Committee |
| NKP Salve and Lata Mangeshkar Hospital |
| North and East Devon Local Research Ethics Committee |
| North and Mid Essex Local Research Ethics Committee |
| North and Mid Hampshire Local Research Ethics Committee |
| North Birmingham Local Research Ethics Committee |
| North Central Wales Local Research Ethics Committee |
| North Cumbria Local Research Ethics Committee |
| North East Wales Local Research Ethics Committee |
| North Manchester Local Research Ethics Committee |
| North Nottinghamshire Local Research Ethics Committee |
| North Sheffield Local Research Ethics Committee |
| North Somerset Local Research Ethics Committee |
| North Staffordshire Local Research Ethics Committee |
| North West Multi-centre Research Ethics Committee |
| North West Surrey Local Research Ethics Committee |
| North West Wales Local Research Ethics Committee |
| Northampton Local Research Ethics Committee |
| Northern and Yorkshire Multi-centre Research Ethics Committee |
| Northumberland Local Research Ethics Committee |
| Norwich Local Research Ethics Committee |
| Nottingham Local Research Ethics Committee |
| Nottingham University Ethics Committee |
| Nuevo Hospital Obregon Ethics Committee |
| Oldham Local Research Ethics Committee |
| Orkney Local Research Ethics Committee |
| Oxfordshire Research Ethics Committee |
| Parkar Medical Foundation Ethics Committee |
| Peterborough and Fenland Local Research Ethics Committee |
| PGIMS Institutional Ethics Committee |
| Phrae Hospital ethical Committee on Human Rights |
| Pinitia Etik Penelitian Kesehatan RSU Dr Soetomo Surabaya |
| Polish Society for Emergency Medicine |
| Poona Medical Research Foundation Ethics Committee |
| Presidency Hospital Ethics Committee |
| Preston, Chorley and South Ribble Local Research Ethics Committee |
| Promotora Medica Las Americas |
| QRG CHRC Ethics Committee |
| R&D Unit, Medical Faculty University of Udayana |
| Rayong Hospital Patient Rights and Ethics Committee |
| Redbridge and Waltham Forest Local Research Ethics Committee |
| Riverside Research Ethics Committee |
| Roi Et Hospital Insitutual Reveiw Board |
| Rotherham Local Research Ethics Committee |
| Royal Free Hospital and Medical School Research Ethics Committee |
| Royal National Orthopaedic Hospital Local Research Ethics Committee |
| Salford and Trafford Local Research Ethics Committee |
| Salisbury and South Wiltshire Research Ethics Committee |
| Sandwell and West Birmingham Local Research Ethics Committee |
| Scarborough and North East Yorkshire Local Research Ethics Committee |
| Sefton Local Research Ethics Committee |
| Shalimar Hospital & Trauma Centre Ethics Committee |
| Shetland Local Research Ethics Committee |
| Shropshire Local Research Ethics Committee |
| Solihull Local Research Ethics Committee |
| South Birmingham Local Research Ethics Committee |
| South Devon Local Research Ethics Committee |
| South East Multi-centre Research Ethics Committee |
| South East Wales Research Ethics Committee Panel D |
| South Essex Local Research Ethics Committee |
| South Humber Local Research Ethics Committee |
| South Manchester Local Research Ethics Committee |
| South Sheffield Research Ethics Committee |
| South Staffordshire Local Research Ethics Committee |
| South Tees Local Research Ethics Committee |
| South West Devon Research Ethics Committee |
| South West Multi-centre Research Ethics Committee |
| South West Surrey Local Research Ethics Committee |
| Southampton and South West Hampshire Research Ethics Committee |
| Southern General Hospital Local Research Ethics Committee |
| Southmead Local Research Ethics Committee |
| Sri Sai Hospital Research Ethics Committee |
| Sri Sakthi Hospital Ethics Committee |
| St Helen’s and Knowsley Local Research Ethics Committee |
| St James Hospital Ethics Committee |
| St Joseph Catholic Health Center of Widikum |
| St Mary's Research Ethics Committee |
| St Stephen's Hospital Ethics Committee |
| St Thomas' Hospital Research Ethics Committee |
| Stockport Local Research Ethics Committee |
| Suffolk Local Research Ethics Committee |
| Sujlam Independent Ethics Committee |
| Sunderland Local Research Ethics Committee |
| Sushrut Hospital |
| Swindon Local Research Ethics Committee |
| Tameside and Glossop Local Research Ethics Committee |
| Tayside Committee on Medical Research Ethics |
| Thames Valley Multi-centre Research Ethics Committee |
| The Medical School, Santou University |
| The Royal Marsden NHS Foundation Trust Local Research Ethics Committee |
| Trent Multi-centre Research Ethics Committee |
| UCLH Research Ethics Committee |
| Universitas Indonesia Fakultas Kedokteran |
| University of Brawijaya Saiful Anwar Hospital, Ethical Committee |
| University Sains Malaysia |
| Usha Hospital Ethics Committee |
| Vadamalayan Ethics Committee |
| Victoria Infirmary Local Research Ethics Committee |
| Wakefield District Research Ethics Committee |
| Walsall Local Research Ethics Committee |
| Wandsworth Local Research Ethics Committee |
| Warwickshire Local Research Ethics Committee |
| West Essex Research Ethics Committee |
| West Hertfordshire Hospitals NHS Trust Research Ethics Committee |
| West Kent Local Research Ethics Committee |
| West Midlands Multi-centre Research Ethics Committee |
| West Somerset Local Research Ethics Committee |
| West Sussex Local Research Ethics Committee |
| Whittington Hospital Local Research Ethics Committee |
| Wolverhampton Local Research Ethics Committee |
| Wrightington, Wigan and Leigh Local Research Ethics Committee |
| York Research Ethics Committee |
| Yorkhill Local Research Ethics Committee |
